# 133. Impact of the BioFire FilmArray Blood Culture Identification 2 (BCID2) Panel on Time to Antimicrobial Escalation or De-escalation in a Community Health System

**DOI:** 10.1093/ofid/ofad500.206

**Published:** 2023-11-27

**Authors:** Katie DelRusso, Jessica Colmerauer, Jennifer Premus

**Affiliations:** Virtua Health, Philadelphia, Pennsylvania; Virtua Health, Philadelphia, Pennsylvania; Virtua Health, Philadelphia, Pennsylvania

## Abstract

**Background:**

The BioFire FilmArray Blood Culture Identification 2 (BCID2) panel is a rapid diagnostic tool that has been shown to decrease time to culture identification and effective therapy in bacteremia, particularly in conjunction with antimicrobial stewardship (AS) participation. The BCID2 panel was introduced at our 5-hospital community health system in May 2021. BCID2 results were relayed to care teams via the standard critical lab process (bedside nurse notified by phone) without notification of the AS team. This analysis compares outcomes after implementation of the BCID2 panel using a standard laboratory notification process.

**Methods:**

This was a retrospective review of adult patients with positive blood cultures at a community health system in New Jersey. Patients with cultures performed using conventional methods (pre-BCID2) 7/1/2021-8/31/2021 were compared to patients with cultures performed using the BCID2 panel (post-BCID2) 7/1/2022-8/31/2022. Exclusion criteria was death within 72 hours, comfort care within 7 days of admission, organism determined to be a contaminant, culture results with a pathogen not on the BCID2 panel, transfer from an outside facility. The primary outcome was time to antimicrobial escalation or de-escalation. Secondary outcomes included time to culture identification, hospital length of stay (LOS), and duration of antimicrobial therapy.

**Results:**

A total of 190 patients were included with 95 patients in each group. Time to de-escalation pre-BCID2 was 59.2 hours versus 35.3 hours post-BCID2 (p < 0.001). Time to culture identification pre-BCID2 was 53.1 hours versus 22.4 hours post-BCID2 (p < 0.001). LOS pre-BCID2 was 12.2 days versus 10.6 days post-BCID2 (p = 0.13). Duration of antimicrobial therapy was 20.2 days pre-BCID versus 18.4 days post-BCID2 (p = 0.29).

**Conclusion:**

Addition of the BCID2 rapid diagnostic panel using a standard critical lab result notification process improved time to antimicrobial escalation or de-escalation by about 24 hours on average. In hospitals with limited stewardship resources, implementation of a rapid diagnostic blood test such as BCID2 without direct stewardship involvement is effective in improving time to antimicrobial escalation or de-escalation.

**Disclosures:**

**All Authors**: No reported disclosures

